# Epigenetic, Genetic, and Functional Germline Alterations of *PAX* Genes in Human Pathology: A Comprehensive Update

**DOI:** 10.3390/cimb48020236

**Published:** 2026-02-23

**Authors:** Valentina Lopez Gomez, Samantha Wegner, Stephanie Ocejo, Dezaray Perez, Diana Jabbour, Virginia Fernandez, Amr Abulaban, Marwan Bahmad, Tarec K. Elajami, Wassim Abou-Kheir, Hisham F. Bahmad

**Affiliations:** 1 Department of Pathology and Laboratory Medicine, University of Miami Miller School of Medicine, Miami, FL 33136, USA;vale371.lg@gmail.com (V.L.G.);; 2 School of Osteopathic Medicine, Campbell University, Buies Creek, NC 27546, USA; 3 Herbert Wertheim College of Medicine, Florida International University, Miami, FL 33199, USA; 4 Faculty of Medical Sciences, Lebanese University, Hadath Campus, Beirut 1003, Lebanon; 5 Division of Vascular Surgery, Department of Surgery, American University of Beirut Medical Center, Beirut 1107, Lebanon; 6 Seymon and Janna Advanced Research Institute, Mount Sinai Medical Center, Division of Cardiology, Columbia University, Miami Beach, FL 33140, USA; tarec.elajami@msmc.com; 7 School of Medicine, New York Medical College, Valhalla, NY 10595, USA; 8 Department of Anatomy, Cell Biology and Physiological Sciences, Faculty of Medicine, American University of Beirut, Beirut 1107, Lebanon

**Keywords:** *PAX*, paired box genes, embryologic development, transcription factors, germline mutations, lineage biomarkers, review

## Abstract

Paired box (*PAX*) genes encode a family of nine transcription factors that function as master regulators of embryogenesis, organogenesis, and lineage specification. Their tightly regulated spatial and temporal expression is essential for the development of multiple organ systems, including the central nervous system, eyes, kidneys, immune system, musculoskeletal system, and endocrine organs. Germline mutations of *PAX* genes result in a broad and often pleiotropic spectrum of human disease, reflecting the developmental programs governed by each family member. Pathogenic variants in *PAX* genes underlie diverse congenital disorders such as aniridia (*PAX6*), renal coloboma syndrome (*PAX2*), otofaciocervical syndrome with immunodeficiency (*PAX1*), Waardenburg syndrome (*PAX3*), maturity-onset diabetes of the young (*PAX4*), and tooth agenesis (*PAX9*). These conditions frequently demonstrate variable expressivity, incomplete penetrance, and overlapping phenotypes, which make it challenging to be clinically recognized. Beyond embryogenesis and embryologic development, emerging evidence indicates that several PAX proteins remain active in postnatal tissue maintenance, adult stem cell regulation, immune function, and regenerative responses (particularly PAX7 in skeletal muscle satellite cells and PAX5 in B-cell homeostasis), further expanding their clinical relevance. This review provides a synopsis of the major, clinically relevant, germline *PAX* gene mutations, emphasizing genotype–phenotype correlations, developmental mechanisms, and disease classification across the organ systems. By integrating molecular genetics with human pathology, we highlight the diagnostic implications of *PAX* genes as central determinants of congenital disease and provide a framework for understanding how alterations in the developmental transcriptional networks translate into human pathology.

## 1. Introduction

**Figure 1:**
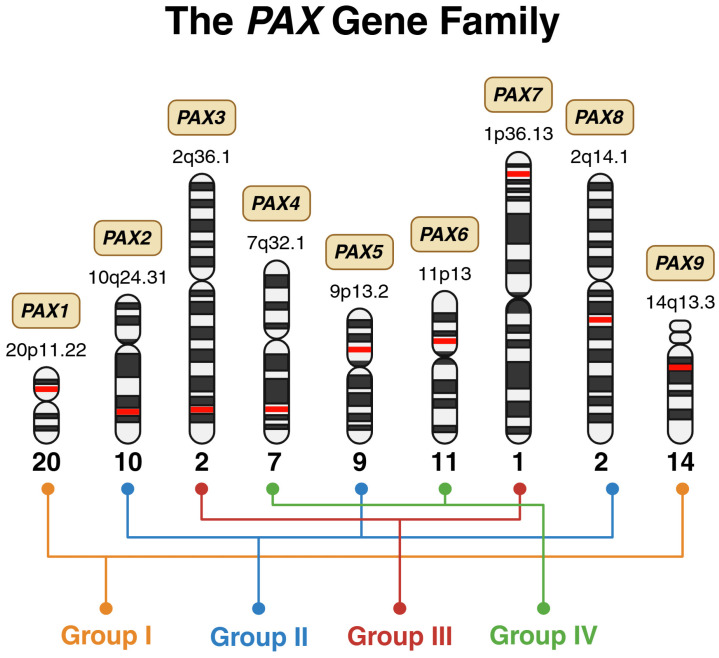
Chromosomal distribution and structural grouping of the human *PAX* gene family. Schematic representation of the nine *PAX* genes mapped to their respective chromosomal loci, illustrated using ideograms of human chromosomes. The precise cytogenetic band for each *PAX* member is indicated adjacent to its chromosomal position, highlighted in red. The *PAX* genes are organized into four structural subclasses: Group I (*PAX1*, *PAX9*), Group II (*PAX2*, *PAX5*, *PAX8*), Group III (*PAX3*, *PAX7*), and Group IV (*PAX4*, *PAX6*). *Created in BioRender. Bahmad, H. (2026)* Available online: https://www.BioRender.com/gv0gwu7 (accessed on 31 January 2026).

Paired box (*PAX*) genes are considered master regulators in both vertebrates and invertebrates, as they govern organ development and are essential for lineage-specific differentiation [1]. Classification within this gene family requires the presence of a paired domain, while the addition of a homeodomain and/or an octapeptide further divides it into four structural subclasses: group I (*PAX1* and *PAX9*), group II (*PAX2*, *PAX5*, and *PAX8*), group III (*PAX3* and *PAX7*), and group IV (*PAX4* and *PAX6*) (Figure 1) [1]. This group of nine transcription factors plays a key role in numerous developmental processes during early growth, as demonstrated in studies of both human and mouse genes [2]. Furthermore, embryogenesis and organogenesis depend heavily on these evolutionarily conserved genes [2]. Despite their crucial role in human development, *PAX* genes are susceptible to mutations like any other gene, with alterations linked to a range of diseases, including lysosomal storage disorders, autoimmune conditions, and cancer [1].

The structural classification of *PAX* genes into four groups carries significant functional and clinical implications beyond mere molecular architecture. This grouping reflects evolutionarily conserved developmental programs and exhibits remarkable topographical coherence in disease manifestation [1,3]. Members within the same *PAX* group tend to cause disorders affecting anatomically related structures, reflecting their shared roles in specific developmental domains. For example, Group I genes (*PAX1* and *PAX9*) both regulate pharyngeal arch derivatives and axial skeletal structures, with mutations causing overlapping phenotypes involving vertebral anomalies, thymic hypoplasia, and craniofacial defects. Group II genes (*PAX2*, *PAX5*, and *PAX8*) share expression in the developing urogenital system and are each associated with renal and/or reproductive tract malformations, though *PAX5* additionally governs B-cell lineage commitment. Group III genes (*PAX3* and *PAX7*) exhibit the most striking functional overlap, both serving as master regulators of neural crest development and skeletal myogenesis; their mutations produce related syndromes involving pigmentary abnormalities, hearing loss, and musculoskeletal defects, while their oncogenic fusions drive myogenic sarcomas. Group IV genes (*PAX4* and *PAX6*), despite structural similarity, have diverged functionally—*PAX6* governing eye and brain development while *PAX4* specializes in pancreatic islet differentiation—yet both influence neuroendocrine lineages. This structure–function relationship provides a practical clinical framework. The affected *PAX* group often predicts the anatomical distribution of disease, aiding differential diagnosis and guiding targeted genetic testing strategies.

*PAX* genes are essential for organogenesis and lineage-specific differentiation, acting as key regulators in organ systems such as the renal, ophthalmic, respiratory, muscular, and central nervous system (CNS) [3]. They are also critical for pattern formation, likely determining the timing and location of organ development. These genes are tightly regulated during organogenesis, and mutations during this period are linked to major developmental defects [4]. Because PAX proteins function through DNA binding, mutations are also associated with an increased risk of cancer development [4].

Group I genes (*PAX1* and *PAX9*) regulate sclerotomes, axial skeletal patterning, and pharyngeal pouch derivatives, including the thymus [5]. *PAX9* specifically maintains tooth structure, and its absence can result in tooth agenesis [6]. Both also contribute to the proliferation of neural crest cells [6]. Group II genes (*PAX2*, *PAX5*, and *PAX8*) are important for the development of the renal system, CNS, and the thyroid gland [7]. Group III genes (*PAX3* and *PAX7*) act as transcription factors for neural crest development, including induction, migration, and cell differentiation [8]. They influence proliferation early in development, determining multiple lineages such as cardiac, sensory, and teeth [8]. Group IV genes (*PAX4* and *PAX6*) are involved in the development of the gastrointestinal and endocrine systems, eyes, and CNS [7]. *PAX* genes are themselves regulated through mechanisms such as posttranslational modifications, degradation, and partner protein interactions, including miRNAs and alternative splicing [7]. Although designed to guide organogenesis and tissue differentiation, mutations in *PAX* genes can result in both neoplastic and non-neoplastic diseases that are debilitating and potentially life-threatening [7].

While prior reviews have addressed *PAX* genes in cancer and developmental disorders, substantial advances have emerged over the past two years that warrant an updated comprehensive review [7,9,10,11]. These include: (i) newly identified germline and somatic *PAX* variants with expanded genotype–phenotype correlations across multiple organ systems; (ii) advances in understanding epigenetic regulation of *PAX* genes, particularly promoter methylation patterns in *PAX1*, *PAX2*, *PAX5*, *PAX6*, and *PAX9* and their roles in tumorigenesis; (iii) emerging roles of PAX proteins in tumor biology and lineage plasticity, including novel fusion partners and metabolic reprogramming mechanisms; (iv) improved diagnostic and molecular testing implications for both germline disorders and somatic alterations [9,12,13,14,15,16,17,18,19,20]. The present review integrates these recent discoveries with established knowledge to provide clinicians and pathologists with a current, comprehensive framework for understanding *PAX*-related human pathology.

## 2. Germline PAX Gene Variations and Functional Consequences

**Figure 2:**
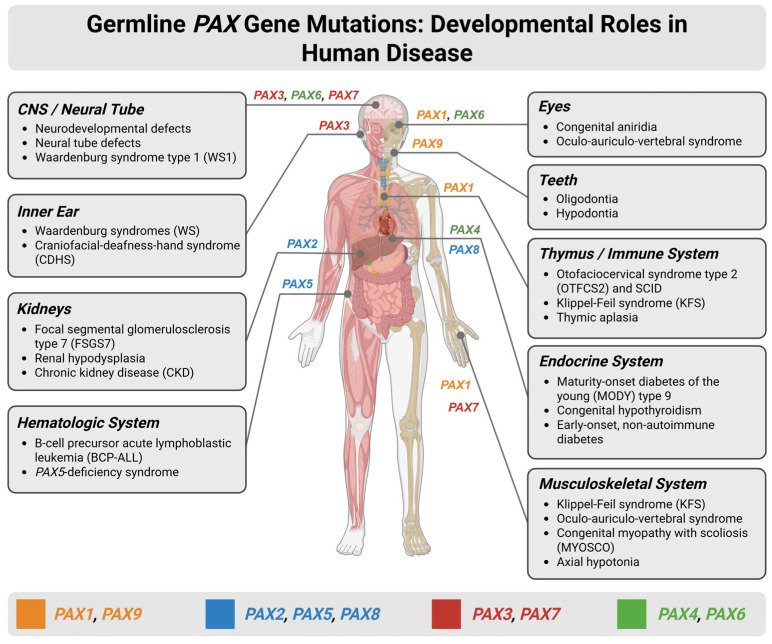
Developmental roles and disease associations of germline *PAX* gene mutations across human organ systems. Schematic overview illustrating the expression patterns of *PAX* genes during human development and the major congenital disorders and disease phenotypes associated with germline mutations. The human body diagram highlights organ-specific involvement of individual *PAX* family members, including the central nervous system (CNS)/neural tube, eyes, inner ear, teeth, thymus and immune system, kidneys, endocrine organs, hematologic system, and musculoskeletal system. Disorders linked to *PAX* gene dysfunction—such as Waardenburg syndromes (WSs), congenital aniridia, renal hypodysplasia, focal segmental glomerulosclerosis, B-cell precursor acute lymphoblastic leukemia, maturity-onset diabetes of the young (MODY), thymic aplasia, and Klippel–Feil syndrome (KFS)—are listed by affected system. WS encompasses four clinical subtypes: WS1 and WS3 result from *PAX3* mutations and feature dystopia canthorum; WS2 (typically *MITF*, *SOX10*, or *SNAI2* mutations) and WS4 (*SOX10*, *EDN3*, or *EDNRB* mutations) lack dystopia canthorum. Inner ear involvement with sensorineural hearing loss occurs across all WS subtypes. *Created in BioRender. Bahmad, H. (2026)* Available online: https://www.BioRender.com/8dixrhs (accessed on 31 January 2026).

Given the diversity of the *PAX* gene family, understanding the pathological spectrum linked to these transcription factors and their variations is essential for elucidating their biological and clinical significance. The various neoplastic and congenital conditions arising from *PAX* dysregulation highlight their central role in developmental biology and tumorigenesis. Here, we systematically examine the major diseases associated with the *PAX* family, including disorders driven by germline mutations, somatic alterations, gene fusions, and epigenetic dysregulation (Figure 2).

Germline mutations can be found through genetic family trees as they are heritable mutations leading to different congenital and neoplastic conditions. Given the distinct developmental functions of each *PAX* gene, germline mutations typically give rise to disorders affecting the same organs and tissues in which that gene normally plays a critical developmental role.

### 2.1. PAX1 Germline Mutations

*PAX1* plays a central role in skeletal and thymic development during embryogenesis. Consequently, germline mutations in this gene can result in a spectrum of developmental disorders involving skeletal malformations and metabolic dysfunction. Consistent with its role in axial skeletogenesis and pharyngeal derivatives, rare germline *PAX1* variants have been described across a spectrum of congenital malformations. In a series of patients with congenital vertebral malformations, Giampietro et al. identified heterozygous missense substitutions in exon 4 of *PAX1* in two males, including one with complex thoracic vertebral defects, rib anomalies, polydactyly, and a ventricular septal defect, suggesting that an altered *PAX1* gene (CCC (Pro) to CTC (Leu) change at amino acid 410) may have contributed to combined axial and cardiac malformations [21]. In Klippel–Feil syndrome, characterized by cervical vertebral fusion, short neck, and low posterior hairline, sequencing of 63 affected individuals uncovered several rare *PAX1* coding and intronic variants not observed in controls, supporting a contribution of *PAX1* haploinsufficiency in at least a subset of cases [22]. Similarly, in two fetuses with Jarcho–Levin syndrome and a “crab-like” thorax, Bannykh et al. demonstrated markedly reduced PAX1 and PAX9 protein expression in vertebral chondrocytes, implicating dysregulated *PAX1*/*PAX9* signaling in severe costovertebral segmentation defects [23]. More recently, biallelic loss-of-function *PAX1* mutations have been shown to underlie autosomal recessive otofaciocervical syndrome type 2 (OTFCS2), a multisystem disorder with craniofacial dysmorphism, vertebral and shoulder-girdle anomalies, thymic aplasia, and profound T-cell immunodeficiency, further delineating the pleiotropic effects of germline *PAX1* disruption [24,25,26] (Table 1).

### 2.2. PAX2 Germline Mutations

*PAX2* is a paired-domain transcription factor that is crucial for normal development of the kidney, urinary tract, and optic nerve, and germline loss-of-function variants underlie a wide spectrum now grouped as *PAX2*-related disorder, classically presenting as renal-coloboma (papillorenal) syndrome (RCS) (Table 2). Heterozygous truncating mutations (nonsense, frameshift, splice-site) clustered in exons 2-4, which encode the paired domain, represent the most common pathogenic variants and typically produce renal hypodysplasia/dysplasia, vesicoureteral reflux, and optic nerve coloboma or dysplasia, with marked intra- and interfamilial variability [29,30,31].

Beyond classic RCS, germline *PAX2* mutations have been increasingly reported in patients with isolated renal disease, including congenital anomalies of the kidney and urinary tract (CAKUT), oligomeganephronia, steroid-resistant nephrotic syndrome, and focal segmental glomerulosclerosis (FSGS). The recurrent paired-domain frameshift c.76dupG (p.Val26fs/Val27fs) is now recognized as the most prevalent *PAX2* pathogenic allele and has been described in families with renal hypodysplasia, RCS, and in twins with FSGS, reflecting the broad phenotypic expressivity of a single variant [20,32,33,39,45]. Larger deletions involving one or more exons, as well as whole-gene deletions, can also cause *PAX2*-related disorder and may present with renal hypodysplasia in the absence of ocular findings [31,41,42,43].

Missense mutations affecting conserved residues in the paired domain generally act as hypomorphic alleles and have been reported in patients with “forme fruste” papillorenal phenotypes or isolated CKD, sometimes without optic nerve anomalies [46,47]. Adult-onset FSGS with little or no structural renal malformation has been linked to heterozygous *PAX2* missense variants that disrupt DNA binding, transactivation, or interactions with corepressors, suggesting both haploinsufficiency and dominant-negative mechanisms [33,37,48,49].

### 2.3. PAX3 Germline Mutations

In humans, germline *PAX3* mutations give rise to Waardenburg syndrome (WS) subtypes and craniofacial-deafness-hand syndrome (CDHS) (Table 3). WS is classified into four clinical subtypes (WS1-4) based on the presence or absence of specific features. WS type 1 (WS1) and WS type 3 (WS3) are both caused by *PAX3* mutations and are characterized by hearing loss, pigmentary disturbances of the hair, skin, and eyes, and dystopia canthorum (lateral displacement of the inner canthi); WS3 (Klein–Waardenburg syndrome) additionally presents with upper limb abnormalities including contractures and hypoplasia. WS type 2 (WS2) lacks dystopia canthorum and is typically caused by mutations in *MITF*, *SOX10*, or *SNAI2* rather than *PAX3*. WS type 4 (WS4 or Waardenburg–Shah syndrome) combines features of WS2 with Hirschsprung disease and results from mutations in SOX10, EDN3, or EDNRB. Sensorineural hearing loss and pigmentary abnormalities affect all WS subtypes, with inner ear involvement (cochlear melanocyte deficiency) representing a common pathogenic mechanism across types [50,51,52,53,54,55,56,57]. Most pathogenic *PAX3* mutations occur within exons 2-6, encoding the paired domain and homeodomain [58,59]. Reports of germline mosaicism and de novo mutations further highlight the variable inheritance patterns [55,60]. CDHS, a related condition, presents with craniofacial anomalies, profound sensorineural deafness, and ulnar deviation with finger contractures, and has been linked to mutations in *PAX3* exon 2 [61].

### 2.4. PAX4 Germline Mutations

*PAX4* encodes a paired-domain transcription factor that is essential for pancreatic islet development and β-cell survival. Germline loss-of-function or hypomorphic *PAX4* variants have been linked to a monogenic form of early-onset diabetes designated maturity-onset diabetes of the young type 9 (MODY9), as well as to increased susceptibility to type 2 diabetes in East Asian populations [72,73,74,75,76,77] (Table 4).

In Thai MODY probands, Plengvidhya et al. identified two rare *PAX4* variants—p.Arg164Trp (R164W) and a splice-site mutation IVS7-1G>A—that were absent in several hundred controls; R164W segregated with diabetes and impaired repression of insulin and glucagon promoters in vitro, while IVS7-1G>A produced aberrant splicing consistent with loss of function. A Japanese MODY family carrying a 39 bp deletion (c.374-412 del39) in exon 3 showed a truncated protein lacking most of the homeodomain, with early-onset, non-autoimmune diabetes in multiple affected relatives [72]. More recently, a heterozygous missense variant c.487C>T in exon 7 was reported in a 19-month-old Chinese boy with MODY9 [77].

Beyond classical MODY, the missense variant p.Arg121Trp (R121W) is enriched in Japanese patients with type 2 diabetes and functionally reduces *PAX4* transcriptional repressor activity [78]. The ethnic-specific variant p.Arg192His (R192H) is associated with earlier age at diabetes onset and increased type 2 diabetes risk [73]. A more recent report describes a novel heterozygous *PAX4* variant c.61C>T (p.Gln21*) in a child with MODY9 and neurodevelopmental impairment, underscoring that *PAX4* dysfunction may extend beyond isolated β-cell failure [79].

### 2.5. PAX5 Germline Mutations

*PAX5* encodes the B-cell-specific activator protein (BSAP), a master regulator of B-cell lineage commitment and maintenance. Germline loss-of-function or hypomorphic *PAX5* variants define a rare, but increasingly recognized, inherited predisposition syndrome that is largely restricted to B-cell precursor acute lymphoblastic leukemia (BCP-ALL), with a separate spectrum of biallelic variants causing immunodeficiency and neurodevelopmental disease (Table 5).

The first description of a *PAX5* leukemia-predisposition allele identified a recurrent heterozygous c.547G>A missense variant (p.Gly183Ser) in the octapeptide domain in two unrelated kindreds with autosomal dominant pre-B-ALL; leukemic blasts consistently showed 9p loss of heterozygosity with retention of the mutant allele, and functional assays demonstrated reduced transactivation capacity, establishing a hypomorphic mechanism [80]. Subsequent reports confirmed germline c.547G>A in additional families and sporadic cases, consolidating this variant as a prototypical *PAX5*-related leukemia predisposition allele with incomplete penetrance [81,82]. Additional germline missense changes at the same hotspot (c.547G>C; p.Gly183Arg) and within the paired domain (c.113G>A; p.Arg38His) have been described in familial BCP-ALL, again behaving as hypomorphic alleles that require secondary somatic events—typically 9p deletion, a second *PAX5* hit, or lesions in JAK-STAT/RAS signaling—for overt leukemogenesis [82,83,84].

More recently, a germline splice-site variant (c.1013-2A>G) resulting in a single-exon deletion of *PAX5* was reported in a child with BCP-ALL, broadening the allelic spectrum to include structural and truncating germline lesions [85]. Aggregated cohort analyses indicate that most *PAX5* leukemia-predisposition alleles are heterozygous, cluster within functional domains, and confer an autosomal dominant syndrome with variable expressivity, in which unaffected carriers are common but at increased lifetime risk of B-lineage malignancy [82].

**Table 5:** Commonly reported pathogenic/likely pathogenic germline *PAX5* variants and their clinical consequences.

Variant	Variant Type and Domain	Zygosity	Main Phenotype/Diagnosis	Inheritance	References
c.547G>A (p.Gly183Ser)	Missense in octapeptide domain (exon 6); hypomorphic allele with reduced transcriptional activity	Heterozygous	Familial pre-B/BCP-ALL; leukemic cells with 9p LOH retaining mutant allele	Autosomal dominant (incomplete penetrance)	[80,81,82]
c.547G>C (p.Gly183Arg)	Missense in octapeptide domain (same codon 183 hotspot); hypomorphic/loss-of-function effect	Heterozygous	Familial BCP-ALL and sporadic pediatric BCP-ALL; often with secondary *9p/PAX5* lesions	Autosomal dominant (incomplete penetrance)	[82,85]
c.113G>A (p.Arg38His)	Missense in paired DNA-binding domain (exon 2); hypomorphic allele with reduced DNA	Heterozygous	Familial BCP-ALL with multiple affected relatives; additional sporadic B-ALL cases	Autosomal dominant leukemia predisposition (incomplete penetrance)	[82,83,84]
c.1013-2A>G (*PAX5* exon 6 deletion)	Canonical splice-acceptor variant at intron 8; predicted in-frame skipping of exon 9 → altered C-terminal region (hypomorphic)	Heterozygous (de novo)	Pediatric BCP-ALL; immunophenotype similar to other *PAX5*-mutated BCP-ALL	Autosomal dominant leukemia predisposition (incomplete penetrance; evidence still limited)	[85,86]

### 2.6. PAX6 Germline Mutations

*PAX6* is a master transcription factor for ocular development, required for proper formation of the optic cup, iris, lens, fovea, and optic nerve, and it remains expressed in select neural and ocular tissues throughout life. Germline *PAX6* variants underlie a broad phenotypic spectrum now grouped as “*PAX6*-related aniridia,” ranging from classic pan-ocular aniridia to isolated foveal hypoplasia, anterior segment dysgenesis (including Peters anomaly), microphthalmia, coloboma, and more complex neurodevelopmental phenotypes [87,88,89,90,91,92,93,94,95,96,97,98,99] (Table 6).

Most pathogenic germline variants are heterozygous loss-of-function changes—nonsense, frameshift, canonical splice-site variants, or multiexonic/whole-gene deletions—that result in *PAX6* haploinsufficiency. These typically cause classic congenital aniridia with high penetrance but variable expressivity. Larger 11p13 deletions that encompass *PAX6* and neighboring *WT1* produce WAGR (Wilms tumor–aniridia–genitourinary anomalies–intellectual disability) syndrome, highlighting the need for cytogenetic or copy-number evaluation in children with aniridia and systemic features [87,91,96].

More than 500 distinct *PAX6* variants have been catalogued, distributed across the paired domain, linker region, homeodomain, and C-terminal proline/serine/threonine-rich (PST) transactivation region. Truncating variants in the DNA-binding domains generally produce typical aniridia, whereas certain missense substitutions in highly conserved residues or in the alternative exon 5a splice region are enriched in atypical phenotypes such as Peters anomaly, anterior segment malformations, or relatively iris-sparing phenotypes (e.g., isolated foveal hypoplasia). Recurrent nonsense and frameshift alleles (e.g., c.265C>T p.Gln89*, c.718C>T p.Arg240*, c.112del p.Arg38Glyfs16, c.278_281del p.Glu93Alafs30) have been reported across multiple cohorts and illustrate the predominance of haploinsufficient mechanisms [70,94,96,98,99,100,101].

Although ocular disease dominates the clinical picture, a subset of individuals with *PAX6* haploinsufficiency or compound heterozygosity exhibit brain malformations (e.g., agenesis of the pineal gland, polymicrogyria), olfactory dysfunction, neurocognitive or psychiatric features, and, rarely, glucose intolerance or diabetes [92,96,102,103].

**Table 6:** Commonly reported pathogenic/likely pathogenic germline *PAX6* variants and their clinical consequences.

Variant	Variant Type and Domain	Zygosity	Main Phenotype/Diagnosis	Inheritance	References
11p13 contiguous deletion including *PAX6* and *WT1*	Large heterozygous deletion of *PAX6* plus neighboring genes at 11p13	Heterozygous	WAGR syndrome (Wilms tumor, aniridia, genitourinary anomalies, intellectual disability)	Usually de novo; autosomal dominant when familial	[91,95,96]
Intragenic multiexon or whole-gene PAX6 deletion	Heterozygous deletion of one or more coding exons or entire gene (often detected by MLPA/CNV analysis)	Heterozygous	Classic aniridia ± foveal hypoplasia, cataract, glaucoma; occasionally neurodevelopmental anomalies	Autosomal dominant; often de novo	[90,91]
c.265C>T (p.Gln89Ter)	Nonsense; premature stop in exon 6 within the paired domain → truncated protein/functional haploinsufficiency	Heterozygous	Congenital aniridia with bilateral congenital cataracts (familial)	Autosomal dominant (familial)	[97]
c.718C>T (p.Arg240*)	Nonsense (premature stop-gain) in exon 9; truncates the homeodomain/linker region	Heterozygous	Classic congenital aniridia with nystagmus; reported in multiple families across ethnicities; often associated with congenital cataract or keratopathy	Autosomal dominant; de novo or familial	[97,98,100]
c.949C>T (p.Arg317*)	Nonsense (premature stop-gain) in exon 8	Heterozygous	Classic/congenital ocular malformation (e.g., Aniridia—pan-ocular: iris hypoplasia/absence, foveal hypoplasia, possible cataract, glaucoma, keratopathy)	Autosomal dominant (haploinsufficiency)	[87,99]
c.781C>T (p.Arg261*)	Nonsense (premature stop-gain) in exon 10	Heterozygous	*PAX6*-related aniridia/eye malformation likely (given truncation and established mutational spectrum)	Autosomal dominant (haploinsufficiency)	[87,99]
c.607C>T (p.Arg203*)	Nonsense (premature stop-gain) in exon 11	Heterozygous	Likely classic aniridia/*PAX6*-associated ocular developmental disorder (consistent with many truncating *PAX6* mutations)	Autosomal dominant (haploinsufficiency)	[87,99]
c.112del (p.Arg38Glyfs*16)	Frameshift deletion in exon 5′ region of the paired domain → premature termination	Heterozygous	Familial aniridia with nystagmus; haploinsufficiency due to truncated paired-domain protein	Autosomal dominant	[100]
c.299G>A (p.Trp100*)	Nonsense mutation in exon 6 within paired domain → early stop	Heterozygous	Congenital aniridia with lens opacities, foveal hypoplasia; classic haploinsufficiency phenotype	Autosomal dominant	[100]
c.278_281del (p.Glu93Alafs*30)	4 bp deletion causing frameshift in paired domain (exon 6) → truncated protein	Heterozygous	Aniridia with spontaneous anterior lens capsule rupture, cataract, and foveal hypoplasia	Autosomal dominant (familial)	[100]
c.76A>G (p.Arg26Gly)	Missense in N-terminal paired domain; affects DNA-binding surface and reduces transcriptional activation	Heterozygous	Iris hypoplasia, variable aniridia, anterior segment dysgenesis, sometimes Peters anomaly	Autosomal dominant; familial	[88,89,101,104]
c.106G>C (p.Gly36Arg)	Missense in PAX6 paired domain; structurally disruptive at a conserved DNA-contact residue	Heterozygous	Anterior segment dysgenesis; iris hypoplasia; reported in families with atypical aniridia	Autosomal dominant	[88,89,93]
c.20T>A, exon 5a (p.Val54Asp)	Missense variant in the alternatively spliced exon 5a affecting N-terminal paired-domain structure	Heterozygous	Anterior segment dysgenesis spectrum, including Peters anomaly, Axenfeld anomaly, congenital cataract, microcornea/microphthalmos, and foveal hypoplasia; variable severity across families	Autosomal dominant (familial or sporadic); possible regional founder effect in Japan	[105]
c.170_174delTGGGC (p.Leu57fs*17)	5 bp deletion in exon 7 → frameshift with premature stop ~17 codons downstream (N-terminal paired domain; predicted loss-of-function, haploinsufficiency via NMD)	Heterozygous	Familial autosomal dominant aniridia with almost complete iris absence, corneal pannus, foveal hypoplasia, markedly reduced visual acuity; in index case, absent pineal gland and severe hypoplasia of anterior commissure on brain MRI	Autosomal dominant (familial)	[92]
c.475delC (p.Arg159fs*47)	Single-base deletion in exon 8 → frameshift with premature stop ~47 codons downstream (paired-domain/central region; predicted loss-of-function, haploinsufficiency via NMD)	Heterozygous	Familial autosomal dominant aniridia with foveal hypoplasia, corneal pannus, anterior/posterior polar cataracts, lens dislocation; in index case, absent pineal gland and absent posterior commissure on brain MRI	Autosomal dominant (familial)	[92]
c.117_128del	In-frame 12 bp deletion in the 5′ coding region of *PAX6* → loss of four amino acids within the paired domain; predicted disruption of DNA binding → loss-of-function	Heterozygous in the father; compound heterozygous in both fetuses	Father: aniridia (sporadic) with midline brain anomalies on CT (aplasia of pineal gland, hypoplasia of corpus callosum and anterior commissure). Fetuses (with both mutations): severe brain malformations, anophthalmia/microphthalmia, agenesis of corpus callosum, massive germinal matrix overgrowth, disorganized cortex and cerebellum, absent pyramidal tracts—lethal PAX6-null-like phenotype	In the father: autosomal dominant *PAX6*-related aniridia/CNS midline defects. In fetuses: effectively autosomal recessive/*PAX6*-null state due to compound heterozygosity.	[103]
c.112del	1 bp deletion near the N-terminal paired domain → frameshift and premature stop; predicted truncated, non-functional protein → loss-of-function	Heterozygous in the mother; compound heterozygous in both fetuses	Mother: aniridia (sporadic) with similar midline brain anomalies on CT (pineal aplasia, corpus callosum and anterior commissure hypoplasia). Fetuses: same severe, early-lethal brain malformation phenotype when combined with c.117_128del12	In the mother: autosomal dominant *PAX6*-related aniridia/CNS anomalies. In fetuses: autosomal recessive/*PAX6*-null phenotype when in trans with c.117_128del	[103]

### 2.7. PAX7 Germline Mutations

*PAX7* encodes a paired-box transcription factor that is indispensable for neural crest formation and maintenance of skeletal muscle satellite cells. Consistent with its role in postnatal myogenesis, human germline *PAX7* variants are now recognized as the cause of a distinctive autosomal recessive “satellite cell-opathy” spectrum, ranging from severe neurodevelopmental syndromes to progressive congenital myopathy with scoliosis (MYOSCO) [106] (Table 7).

The first human *PAX7*-related disorder was described by Proskorovski-Ohayon et al., who reported a consanguineous family with an autosomal recessive syndrome of failure to thrive, severe global developmental delay, microcephaly, axial hypotonia, pyramidal signs, dystonia, seizures, irritability, and self-mutilation due to a biallelic splice-disrupting mutation in *PAX7* isoform 3 [107].

Subsequently, Feichtinger et al. identified biallelic loss-of-function *PAX7* variants (nonsense, frameshift, and splice-site changes) in multiple unrelated families with progressive congenital myopathy, axial and limb-girdle weakness, early scoliosis, ptosis, and markedly reduced satellite cell numbers on muscle biopsy, establishing *PAX7*-associated MYOSCO as a primary satellite cell disorder [108]. More recent work has expanded the clinical and imaging spectrum of *PAX7*-related myopathy, describing additional biallelic missense variants with characteristic whole-body muscle MRI patterns and variable scoliosis severity [109,110].

Beyond myopathy, rare heterozygous and compound heterozygous *PAX7* variants have been reported in cohorts with congenital scoliosis, suggesting that impaired *PAX7*-mediated muscle development and paraspinal muscle dysfunction may contribute to vertebral malformations in at least a subset of cases [111].

**Table 7:** Commonly reported pathogenic/likely pathogenic germline *PAX7* variants and their clinical consequences.

Variant	Variant Type and Domain	Zygosity	Main Phenotype/Diagnosis	Inheritance	References
Splice-site variant in *PAX7* isoform 3	Predicted loss-of-function splice-site change leading to aberrant splicing and reduced PAX7 protein; affects DNA-binding/activation of target genes	Homozygous	Severe neurodevelopmental syndrome with failure to thrive, microcephaly, axial hypotonia, pyramidal signs, dystonia, seizures, irritability, and self-mutilation	Autosomal recessive	[107]
Multiple biallelic truncating and splice-site variants across *PAX7* (nonsense, frameshift, canonical splice; distributed across paired and C-terminal transactivation domains)	Loss-of-function variants causing markedly reduced or absent PAX7, satellite cell depletion, and impaired myogenesis	Mostly homozygous; some compound heterozygous	Progressive congenital myopathy with scoliosis: early-onset axial and limb-girdle weakness, ptosis, progressive scoliosis, restrictive respiratory failure; muscle biopsies show reduced satellite cells and dystrophic changes	Autosomal recessive	[108]
Novel biallelic *PAX7* variants in congenital myopathy with scoliosis	Missense/splice-affecting variants; functionally consistent with *PAX7* loss-of-function; characteristic whole-body MRI pattern	Homozygous	*PAX7*-related congenital myopathy with prominent axial weakness, early scoliosis, and distinctive whole-body muscle MRI	Autosomal recessive	[109]
Multiple rare heterozygous *PAX7* missense variants enriched in congenital scoliosis cohort	Predicted deleterious missense variants, many clustering within or near DNA-binding domains	Heterozygous (in most probands; some complex inheritance)	Congenital scoliosis (vertebral formation/segmentation defects) with or without neuromuscular features; *PAX7* variants proposed as risk alleles contributing to congenital scoliosis pathogenesis via impaired muscle development	Likely autosomal dominant or risk-modifying; segregation incomplete	[111]

### 2.8. PAX8 Germline Mutations

*PAX8* is a paired-domain transcription factor essential for thyroid organogenesis, as well as kidney and Müllerian tract development. Monoallelic loss-of-function variants in *PAX8* are a well-established cause of congenital hypothyroidism due to thyroid dysgenesis, typically presenting with thyroid hypoplasia or ectopy (Table 8). Most pathogenic germline variants are missense, nonsense, or small indels clustered within the N-terminal paired domain (amino acids 9-133), where they impair DNA binding and transcriptional activation of thyroid-specific targets such as thyroglobulin and thyroperoxidase [112,113,114].

Early reports described heterozygous paired-domain substitutions (for example p.Arg31His, p.Gln40Pro, p.Ser54Gly, p.Cys57Tyr, p.Leu62Arg) that segregate with familial congenital hypothyroidism and show loss of transactivation in vitro, supporting a haploinsufficiency model with variable penetrance and intrafamilial expressivity [112,113,114,115]. Subsequent cohort studies across different ethnicities confirmed that pathogenic *PAX8* variants account for roughly 1-3% of congenital hypothyroidism cases due to thyroid dysgenesis, with phenotypes ranging from overt neonatal hypothyroidism with severe hypoplasia to subclinical disease with a normal-appearing gland in situ [116,117,118,119].

Although *PAX8* is also expressed in the kidney and urogenital tract, extrathyroid malformations are relatively uncommon, occurring in <10% of carriers; reported anomalies include renal agenesis, vesicoureteral reflux, and other CAKUT-spectrum defects [114,120]. More recent work has expanded the mutational spectrum beyond the paired domain to include truncating variants in the transactivation domain (p.Leu186Hisfs*22) and in-frame deletions (p.Glu90del) that retain partial transcriptional activity yet still cause congenital hypothyroidism with a gland in situ [116,118,119].

**Table 8:** Commonly reported pathogenic/likely pathogenic germline *PAX8* variants and their clinical consequences.

Variant	Variant Type and Domain	Zygosity	Main Phenotype/Diagnosis	Inheritance	References
c.92G>A (p.Arg31His)	Missense in paired domain (DNA-binding)	Heterozygous	Congenital hypothyroidism with thyroid dysgenesis (ectopy or hypoplasia)	Autosomal dominant	[112,117]
c.119A>C (p.Gln40Pro)	Missense in paired domain	Heterozygous	Congenital hypothyroidism with thyroid hypoplasia; mother with normal-sized gland and mild adult-onset autoimmune hypothyroidism	Autosomal dominant	[113]
c.160A>G (p.Ser54Gly)	Missense in paired domain helix	Heterozygous	Congenital hypothyroidism with normal-sized eutopic thyroid at birth; one carrier with unilateral renal agenesis	Autosomal dominant	[114]
c.170G>A (p.Cys57Tyr)	Missense in paired domain	Heterozygous	Familial congenital hypothyroidism with thyroid hypoplasia	Autosomal dominant	[115]
c.185T>G (p.Leu62Arg)	Missense in paired domain	Heterozygous	Congenital hypothyroidism with thyroid dysgenesis (ectopy/hypoplasia)	Autosomal dominant	[112,121]
c.322C>T (p.Arg108*)	Nonsense, truncating within paired domain	Heterozygous	Congenital hypothyroidism with thyroid hypoplasia	Autosomal dominant	[112,120]
c.74C>G (p.Pro25Arg)	Missense near N-terminus/paired domain boundary	Heterozygous	Congenital hypothyroidism with thyroid hypoplasia across three generations; some carriers with urogenital malformations	Autosomal dominant	
c.116A>C (p.His39Pro)	Missense in paired domain	Heterozygous	Familial congenital hypothyroidism with thyroid hypoplasia	Autosomal dominant	[117,118]
c.122G>T (p.Gly41Val)	Missense in paired domain	Heterozygous	Congenital hypothyroidism with thyroid agenesis or small eutopic gland	Autosomal dominant	
c.280G>A (p.Asp94Asn)	Missense in paired domain	Heterozygous	Congenital hypothyroidism with thyroid dysgenesis; often gland in situ	Autosomal dominant	[116,117]
c.268_270delGAA (p.Glu90del)	In-frame deletion in paired domain	Heterozygous	Congenital hypothyroidism with gland in situ	Autosomal dominant	[116]
c.555dupA (p.Leu186Hisfs*22)	Frameshift truncation	Heterozygous	Congenital hypothyroidism with gland in situ	Autosomal dominant	[116]

### 2.9. PAX9 Germline Mutations

*PAX9* encodes a paired-box transcription factor essential for tooth morphogenesis, particularly posterior tooth development. Germline loss-of-function and missense variants in *PAX9* are among the most frequent monogenic causes of non-syndromic tooth agenesis (selective tooth agenesis 3, STHAG3), usually presenting as autosomal dominant hypodontia or oligodontia with a molar-predominant pattern (Table 9).

The first description of *PAX9*-related oligodontia identified heterozygous truncating variants segregating with familial absence of posterior teeth, establishing the concept of *PAX9* haploinsufficiency. Subsequent case series and functional studies have expanded the mutational spectrum to include nonsense, frameshift, initiation codon, and paired-domain missense variants, as well as regulatory changes, with 50–60 distinct pathogenic or likely pathogenic alleles now catalogued.

Systematic reviews and genotype–phenotype analyses consistently show that null (nonsense/frameshift) variants are associated with more severe oligodontia, particularly involving second molars, whereas some missense substitutions within the paired domain allow partial protein function and milder phenotypes. Rare large deletions encompassing *PAX9* further support dosage sensitivity. Although most reported cases are non-syndromic, occasional families show co-occurring craniofacial or dental anomalies, suggesting possible interaction with other odontogenic pathways (e.g., *MSX1*, *WNT* signaling).

## 3. Somatic PAX Gene Variations and Functional Consequences

Across human cancers, *PAX* genes are recurrently altered at the somatic level, but the mode of alteration varies substantially between the nine family members. Rather than classic hotspot missense mutations in all genes, the landscape includes promoter hypermethylation (*PAX1*, *PAX6*, *PAX9*), copy-number gain and overexpression (*PAX2*, *PAX8*, *PAX9*), oncogenic gene fusions (*PAX3*, *PAX7*, *PAX5*, *PAX8*), and true coding “driver” mutations.

*PAX1* behaves primarily as a tumor-suppressor gene in squamous epithelia. Dense promoter CpG island hypermethylation and transcript silencing are frequent in high-grade squamous intraepithelial lesions (HSIL) of the cervix (cervical intraepithelial neoplasia; CIN) and invasive cervical carcinoma [9,12,16], as well as in head-and-neck and oral squamous cell carcinomas, and correlate with higher grade and poorer outcome [13,146,147].

*PAX2* is rarely mutated at the coding level in solid tumors, but copy-number gain and strong nuclear expression have been reported in renal cell carcinoma and subsets of ovarian and endometrial carcinomas, where *PAX2* acts as a lineage-restricted survival factor and may cooperate with *PAX8* [10,148,149,150,151]. In addition, although PAX2 loss is a frequent immunophenotypic abnormality in AH/EIN—including within endometrial polyps, where PAX2 loss occurs in approximately two-thirds of cases (64.8%)—this aberrancy reflects transcriptional silencing rather than coding mutations, underscoring that *PAX2* inactivation in endometrial neoplasia may be epigenetic or regulatory rather than mutational in origin [152].

*PAX3* and *PAX7* are prototypically altered through balanced translocations that fuse their N-terminal paired domain to the C-terminal transactivation domain of *FOXO1* (*PAX3*::*FOXO1*, *PAX7*::*FOXO1*) in alveolar rhabdomyosarcoma [153,154], and to *MAML3* (*PAX3*::*MAML3*) as well as *NCOA1*, *NCOA2*, *FOXO1*, and *WWTR1* (*PAX3*::*NCOA1* associated with rhabdomyoblastic differentiation, *PAX3*::*NCOA2*, *PAX3*::*FOXO1*, and *PAX3*::*WWTR1*) in biphenotypic sinonasal sarcoma [155,156,157,158,159,160].

For *PAX4*, most pathogenic variants are germline alleles predisposing to MODY9 and type 2 diabetes, discussed in the previous section. *PAX5* is one of the most frequently altered genes in B-cell precursor acute lymphoblastic leukemia (BCP-ALL) [161]. Approximately 40% of pediatric BCP-ALL cases harbor *PAX5* variants, including point mutations, intragenic deletions, and fusions [17,162]. A single recurrent missense change, p.Pro80Arg (c.239C>G), defines a distinct molecular subtype (“*PAX5*^P80R^”) with characteristic gene-expression profile and biallelic PAX5 inactivation [163]. *PAX5* is also affected by fusions such as *PAX5*::*JAK2* which exerts a distinct gene expression signature in B-ALL and is exclusively found in the Ph-like subtype [164,165,166].

*PAX6* acts as a tumor suppressor in the body. In glioblastoma, non-small lung cancer, and liver cancer, reduced *PAX6* expression—often associated with promoter methylation—is linked to higher grade and shorter survival, as demonstrated by in vitro and in vivo studies [15,102,167,168,169]. Somatic *PAX6* mutations—mostly truncating or paired-domain missense—have also been reported in colorectal and pancreatic cancers [170,171,172].

*PAX8* is a lineage-defining factor in thyroid, renal, and Müllerian carcinomas [173]. Somatically, it is altered by (i) *PAX8*::*PPARγ* and related fusions in follicular thyroid carcinoma [174]; (ii) non-coding mutations in distal regulatory elements that enhance *PAX8* binding and drive proliferation in ovarian and renal cancer models [175,176,177,178]; (iii) a documented paired-domain missense mutation p.Arg49His (*PAX8*^R49H^) in intramucosal gastric carcinoma with lymph-node metastasis [179].

Lastly, *PAX9* is frequently altered in squamous carcinomas. In esophageal squamous cell carcinoma, reduced *PAX9* expression via promoter methylation or copy-number loss is associated with poor prognosis and reduced radiosensitivity [180,181,182], whereas in lung squamous cell carcinoma *PAX9* resides within a 14q13.3 amplicon (with *NKX2-1*/*NKX2-8*) and appears to support tumor growth as a lineage-dependency factor [183,184].

## 4. Epigenetic PAX Gene Modifications

Epigenetic regulation of *PAX* genes represents a critical mechanism of transcriptional control in both normal development and disease pathogenesis. Unlike genetic mutations that alter DNA sequence, epigenetic modifications (including DNA methylation, histone modifications, and chromatin remodeling) reversibly modulate *PAX* gene expression and contribute to diverse pathological processes [2].

### 4.1. DNA Methylation of PAX Gene Promoters

Aberrant promoter hypermethylation is the most extensively characterized epigenetic alteration affecting *PAX* genes in human cancer. *PAX1* promoter methylation occurs frequently in CIN3 and invasive cervical carcinoma, where specific CpG sites (notably cg16767801) serve as biomarkers for disease progression and screening triage in high-risk HPV-positive women [14]. Similarly, *PAX1* methylation is observed in oral and head-and-neck squamous cell carcinomas, correlating with higher tumor grade and poorer outcomes [13].

*PAX2* exhibits context-dependent epigenetic regulation. In endometrial cancer, a distinct mechanism involving replacement of active chromatin marks (H3K27ac, H3K4me3) with repressive marks (H3K27me3) silences PAX2 expression in more than 80% of cases, representing a novel “third mechanism” of tumor suppressor inactivation beyond mutation and promoter methylation [185]. Conversely, in some endometrial cancers, *PAX2* promoter hypermethylation paradoxically promotes PAX2 expression by silencing the repressive transcription factor MZF1, illustrating the complexity of epigenetic regulation [186,187]. *PAX2* methylation patterns also distinguish subtypes of renal cell carcinoma, with differential methylation observed in clear cell, papillary, and oncocytic variants [188].

*PAX5* promoter methylation of both alpha and beta isoforms occurs in approximately 65% of breast and lung cancers, with dense methylation correlating with transcriptional silencing [189]. *PAX5β* encodes B-cell-specific activator protein (BSAP), which directly regulates CD19 (a negative regulator of cell growth) suggesting that *PAX5* methylation contributes to neoplastic development through loss of growth control [189,190]. In non-small cell lung cancer, *PAX5* methylation is associated with silencing and loss of its tumor-suppressor functions, including inhibition of β-catenin signaling and upregulation of GADD45G [190].

*PAX6* promoter hypermethylation has been documented in glioblastoma, non-small cell lung cancer, and hepatocellular carcinoma, where it correlates with reduced expression, higher tumor grade, and shorter survival [15]. Experimental restoration of PAX6 expression through demethylation inhibits tumor growth and metastasis, confirming its tumor-suppressor role [15].

*PAX9* methylation is frequent in esophageal squamous cell carcinoma, where it associates with poor prognosis and reduced radiosensitivity [191].

### 4.2. Histone Modification and Chromatin Remodeling

Beyond DNA methylation, *PAX* genes recruit chromatin-remodeling complexes that introduce activating or repressive histone marks [2]. *PAX2* silencing in endometrial cancer involves spreading of repressive H3K27me3 marks in a “pearl necklace” pattern dictated by three-dimensional genome organization and cohesin loops, preventing transcriptional dysregulation of neighboring genes [185]. *PAX7* exhibits pioneer transcription factor activity, uniquely capable of “opening” closed chromatin to initiate developmental programs [192]. In parathyroid adenomas, reduced histone H3K9 acetylation at the *PAX1* promoter contributes to transcriptional downregulation [193].

### 4.3. Regulatory Feedback Loops

Epigenetic regulation of *PAX* genes often involves complex feedback mechanisms. In breast cancer, a *PAX5*-*miR*-*142*-*DNMT1*/*ZEB1* negative feedback loop regulates tumor progression. *PAX5* positively regulates *miR-142-5p/3p*, which in turn targets *DNMT1* and *ZEB1*; these factors then promote *PAX5* promoter methylation, creating a self-reinforcing circuit [194].

### 4.4. Clinical and Therapeutic Implications

The reversibility of epigenetic modifications makes them attractive therapeutic targets. Treatment with demethylating agents (5-aza-2′-deoxycytidine) restores *PAX* gene expression in multiple cancer models, suggesting potential for epigenetic therapies [187,189,190]. Furthermore, *PAX* gene methylation status shows promise as a diagnostic and prognostic biomarker, particularly *PAX1* methylation in cervical cancer screening and *PAX2* methylation patterns in endometrial and renal neoplasms.

## 5. Conclusions

The paired box (*PAX*) gene family occupies a central position at the intersection of developmental biology and human disease. Through highly conserved, lineage-defining transcriptional programs, PAX proteins orchestrate organogenesis, cellular differentiation, and tissue patterning across multiple systems, including the nervous, musculoskeletal, endocrine, immune, renal, and sensory organs. Germline alterations of these tightly regulated developmental pathways give rise to a broad spectrum of congenital and inherited disorders whose phenotypes closely mirror the embryologic roles of each *PAX* gene. The resulting conditions—ranging from aniridia, renal coloboma syndrome, and Waardenburg syndrome to otofaciocervical syndrome, congenital immunodeficiency, monogenic diabetes, and tooth agenesis—underscore the profound and pleiotropic consequences of *PAX* dysregulation.

From a pathology perspective, understanding germline *PAX* gene mutations provides critical insights into genotype–phenotype correlations, disease pathogenesis, and developmental origins of human pathology. Although PAX proteins are widely recognized for their diagnostic utility in surgical pathology through immunohistochemistry and molecular testing, their roles as drivers of congenital disease and tissue-specific vulnerability are equally important. Increasing recognition of variable expressivity, incomplete penetrance, and overlapping phenotypes further emphasizes the need for integrated clinicopathologic and genomic approaches when evaluating patients with suspected *PAX*-related disorders.

Recent advances have illuminated the multifaceted roles of *PAX* genes beyond classical germline disorders. Epigenetic silencing through promoter methylation and histone modifications represents a prevalent mechanism of *PAX* gene inactivation in cancer, offering reversible therapeutic targets [185,189]. Somatic *PAX* gene fusions (particularly *PAX3/7::FOXO1* in rhabdomyosarcoma and *PAX5::JAK2* in B-cell leukemia) define distinct molecular subtypes with specific therapeutic vulnerabilities [195]. Furthermore, the recognition that PAX proteins maintain adult stem cell populations and regulate tissue regeneration suggests broader roles in aging, tissue repair, and regenerative medicine [196].

Beyond development, studies suggest that select *PAX* genes remain active in postnatal tissue homeostasis, immune regulation, and regeneration, raising important questions about their broader biological functions and long-term disease implications. As genomic sequencing becomes increasingly embedded in clinical practice, systematic characterization of germline *PAX* variants will refine diagnostic precision, improve genetic counseling, and inform surveillance strategies. Continued integration of developmental genetics with human pathology will be essential to fully elucidate the lifelong impact of *PAX* gene dysregulation and to translate these insights into improved patient care.

## Figures and Tables

**Table 1:** Commonly reported pathogenic/likely pathogenic germline *PAX1* variants and their clinical consequences.

Variant	Variant Type and Domain	Zygosity	Main Phenotype/Diagnosis	Inheritance	References
c.1104C>A (p.Cys368*)	Truncating (nonsense; null allele) mutation	Homozygous	OFCS type 2 (OTFCS2) with severe combined immunodeficiency (SCID)	Autosomal recessive	[24]
c.1173_1174insGCCCG (p.Pro392Alafs*19)	5 bp frameshift insertion in exon 4 → premature stop, truncating C-terminus	Homozygous	OTFCS2 with thymic aplasia	Autosomal recessive	[25]
c.1212dup (p.Gly405Argfs*51)	Single-base duplication at amino acid position 405 in exon 4 → frameshift and premature stop	Homozygous	OTFCS2 with SCID	Autosomal recessive	[27]
c.1154_1157dup (p.Tyr386*)	Four base duplication in exon 4 → stop-gain/truncation → nonsense/truncation (null) mutation	Heterozygous	Oculo-auriculo-vertebral syndrome (OAVS)	Autosomal dominant	[28]
Multiple rare *PAX1* coding variants (various missense/small indels)	Mostly heterozygous missense variants of uncertain/variable functional impact	Heterozygous (in most reported individuals)	Klippel–Feil syndrome (KFS) and related vertebral segmentation defects	Likely autosomal dominant (KFS) in most families	[5,22]

**Table 2:** Commonly reported pathogenic/likely pathogenic germline *PAX2* variants and their clinical consequences.

Variant	Variant Type and Domain	Zygosity	Main Phenotype/Diagnosis	Inheritance	References
c.76dupG (p.Val26Glyfs*28)	Single-base insertion in exon 2 → early frameshift beginning at Val26, introducing a premature termination codon 28 amino acids downstream; paired domain; predicted null allele	Heterozygous	Classic RCS: bilateral renal hypodysplasia/dysplasia, vesicoureteral reflux, optic nerve coloboma/dysplasia; also reported in focal segmental glomerulosclerosis type 7 (FSGS7) with congenital anomalies of the kidney and urinary tract (CAKUT)	Autosomal dominant (often de novo)	[20,32,33]
c.76delG (p.Val26fs)	Single-base deletion in exon 2 → early frameshift and truncation; paired domain; loss-of-function	Heterozygous	RCS with optic disc dysplasia; variable renal hypodysplasia and chronic kidney disease (CKD)	Autosomal dominant (familial and de novo)	[32,34,35]
c.343C>T (p.Arg115*)	Nonsense mutation in exon 4 (paired domain) → truncated protein; null allele	Heterozygous	Classic RCS with optic nerve dysplasia, renal hypodysplasia; also reported in isolated FSGS with bilateral renal hypodysplasia	Autosomal dominant	[20,31,35,36]
c.310C>T (p.Arg104*)	Nonsense in exon 4 (paired domain) → truncated protein	Heterozygous	*PAX2*-related disorder: RCS/CAKUT with FSGS, with or without optic nerve anomalies	Autosomal dominant	[19,20,37]
c.418C>T (p.Arg140Trp/p.Arg140Ile)	Missense change in paired domain affecting DNA-binding interface; partial loss-of-function	Heterozygous	FSGS7 with bilateral kidney hypodysplasia or isolated proteinuria; sometimes within broader *PAX2*-related disorder	Autosomal dominant	[20,37,38]
c.565G>A (p.Gly189Arg)	Missense in transactivation domain; functionally hypomorphic	Heterozygous	Adult-onset FSGS7 with bilateral pelvic dilatation or other mild CAKUT; variable CKD progression	Autosomal dominant (familial and sporadic)	[20,37]
c.392delG (p.Ser131Thrfs*28)	Single-base G deletion of nucleotide 935 in exon 3 → frameshift mutation; paired domain	Heterozygous (often de novo)	Severe fetal/infantile renal hypodysplasia with or without optic nerve anomalies; may present as antenatal oligohydramnios and early ESRD	Autosomal dominant (de novo in many cases)	[31,39,40]
c.619_624dup (p.Glu194_Thr195dup)	In-frame hexanucleotide duplication in the paired-domain of *PAX2*; predicted to impair DNA binding and transactivation	Heterozygous	RCS with optic disc anomalies and renal hypoplasia; intrafamilial variability in severity	Autosomal dominant	[29]
Whole-gene or multi-exon *PAX2* deletions (10q24 microdeletions)	Heterozygous contiguous-gene or intragenic deletions involving *PAX2*; haploinsufficiency	Heterozygous	*PAX2*-related disorder with renal hypodysplasia, CKD/ESRD; optic nerve anomalies may be absent; additional features depend on size of deletion	Autosomal dominant (often de novo)	[31,41,42,43]
Multiple truncating and missense variants across exons 2-4 and downstream exons	Nonsense, frameshift, splice-site, and pathogenic missense variants, particularly clustered in the paired domain; most predicted loss-of-function	Mostly heterozygous; rare mosaicism	*PAX2*-related disorder spectrum, ranging from classic RCS to isolated CAKUT, isolated optic nerve anomalies, or familial FSGS; marked intra- and interfamilial variability	Autosomal dominant	[20,31,32,33,44]

**Table 3:** Commonly reported pathogenic/likely pathogenic germline *PAX3* variants and their clinical consequences.

Variant	Variant Type and Domain	Zygosity	Main Phenotype/Diagnosis	Inheritance	References
c.626C>A (p.Ser209*)	Nonsense (stop-gain) in coding region upstream of homeodomain → premature stop → truncated protein lacking entire homeodomain and transactivation domain → loss-of-function	Heterozygous	Waardenburg syndrome type 1 (WS1) with dystopia canthorum, hearing loss, and pigmentary changes	Autosomal dominant	[62]
c.667C>T (p.Arg223*)	Nonsense (stop-gain) in central region → premature stop → truncated protein lacking the C-terminal transactivation domain → loss-of-function	Heterozygous	WS1 with typical auditory-pigmentary features	Autosomal dominant	[62]
c.209G>A (p.Cys70Tyr)	Missense mutation in the paired domain (exon 2); substitution of fully conserved Cys70 → Tyr; disrupts paired-domain structure and predicted DNA-binding; high Grantham score (194) → radical physicochemical change; likely loss-of-function	Heterozygous	WS1 with congenital sensorineural hearing loss and patchy depigmentation of hair/skin	Autosomal dominant	[63]
c.788T>G (p.Val263Gly)	Missense in homeodomain (helix 3) affecting DNA-binding surface	Heterozygous	Familial WS1 with intrafamilial variability (dystopia canthorum, striking blue irides, hearing loss)	Autosomal dominant	[64]
c.1107C>G (p.Ser369Arg)	Missense variant in the C-terminal transactivation domain of *PAX3* → Alters a conserved residue within the transactivation domain → predicted impairment of *PAX3* transcriptional activity (non-null, hypomorphic effect)	Heterozygous	WS1 in a Japanese proband, presenting with: heterochromic iris, unilateral hearing loss, synophrys, cleft lip, and cryptorchidism	Autosomal dominant	[65]
c.788dup (p.Gln264ThrfsTer5)	Single-base duplication → frameshift beginning at Gln264 in the homeodomain—adjacent region (near C-terminal end of the homeodomain) → premature termination 5 codons downstream → truncated protein lacking much of the transactivation domain → loss-of-function	Heterozygous	WS1 in Chinese Yugur family, with iris hypopigmentation, sensorineural hearing loss, and dystopia canthorum	Autosomal dominant	[66]
c.384del13 (p.Gly128fs)	Multi-exonic deletions in exon 3 encompassing part of the paired domain → haploinsufficiency	Heterozygous	WS3 (Klein–Waardenburg) with limb contractures and upper-limb involvement in addition to classic WS1 features	Autosomal dominant	[67,68,69]
c.139A>G (p.Asn47Lys)	Missense substitution in the paired domain (exon 2) → alters highly conserved residue; disrupts DNA-binding specificity and protein function	Heterozygous	Craniofacial-deafness-hand syndrome (CDHS) with craniofacial dysmorphism, severe deafness, and hand contractures	Autosomal dominant	[61]
Multiple missense, nonsense, splice-site and frameshift variants in exons 2-6 (paired domain + homeodomain)	Diverse loss-of-function and missense changes affecting DNA-binding and/or transactivation	Mostly heterozygous	WS1 and WS3 across multiple ethnic groups; variable expressivity of hearing loss, pigmentary changes, and limb anomalies; includes de novo and familial variants	Autosomal dominant	[55,59,70,71]

**Table 4:** Commonly reported pathogenic/likely pathogenic germline *PAX4* variants and their clinical consequences.

Variant	Variant Type and Domain	Zygosity	Main Phenotype/Diagnosis	Inheritance	References
c.361C>T (p.Arg121Trp)	Missense in paired domain; reduces repressor activity on β-cell promoters	Heterozygous	Early-onset/familial type 2 diabetes in Japanese; non-autoimmune, β-cell-predominant defect	Autosomal dominant familial clustering	[11,78]
c.490C>T (p.Arg164Trp)	Missense in paired domain; impairs repression of insulin and glucagon promoters	Heterozygous	Maturity-onset diabetes of the young (MODY)-like diabetes in Thai family; early-onset, non-autoimmune	Segregates in autosomal dominant fashion within family	[74,76]
IVS7-1G>A	Canonical splice-acceptor mutation at intron 7; causes aberrant mRNA splicing and predicted loss of function	Heterozygous	MODY type 9 (MODY9) with severe diabetic complications in extended Thai family	Autosomal dominant with high penetrance	[76]
c.374_412del (p.Thr125_Glu137del)	39 bp in-frame deletion removing part of the paired-type homeodomain; truncation of C-terminal region	Heterozygous	MODY9 in Japanese family; early-onset, non-autoimmune diabetes affecting multiple generations	Autosomal dominant	[72]
c.487C>T (p.Arg163Trp)	Missense variant in homeodomain region; predicted damaging by in silico tools	Heterozygous	MODY9 in a 19-month-old Chinese boy with insulin-dependent diabetes and negative autoantibodies	Suspected autosomal dominant (sporadic case)	[77]
c.575G>A (p.Arg192His)	Missense in homeodomain; reduces repression of β-cell genes and alters β-cell survival under glucotoxic stress	Heterozygous	MODY-like diabetes and earlier age at onset of type 2 diabetes; common risk allele in East Asians	Multifactorial; susceptibility allele rather than classic Mendelian MODY	[73]
c.61C>T (p.Gln21*)	Nonsense mutation in exon 1 → premature stop codon at amino acid 21; predicted early truncation eliminating paired domain and full-length protein (null allele)	Heterozygous	MODY9 in a child with insulin-dependent, non-autoimmune early-onset diabetes; case also associated with neurodevelopmental impairment	Likely autosomal dominant; single sporadic case reported	[79]

**Table 9:** Commonly reported pathogenic/likely pathogenic germline *PAX9* variants and their clinical consequences.

Variant	Variant Type and Domain	Zygosity	Main Phenotype/Diagnosis	Inheritance	References
c.340A>T (p.Lys114Ter)	Nonsense; truncation in paired domain	Heterozygous	Non-syndromic molar oligodontia (posterior-predominant missing teeth)	Autosomal dominant	[122,123]
c.62T>C (p.Leu21Pro)	Missense in N-terminal paired domain	Heterozygous	Familial oligodontia with posterior tooth agenesis	Autosomal dominant	[124,125]
c.76C>T (p.Arg26Trp)	Missense in paired domain helix–turn–helix motif	Heterozygous	Non-syndromic oligodontia (molar-predominant)	Autosomal dominant	[126,127,128]
c.151G>A (p.Gly51Ser)	Missense in paired domain; reduces DNA-binding	Heterozygous	Non-syndromic oligodontia with variable posterior tooth loss	Autosomal dominant	[124,129,130,131]
c.218dup (p.Ser74fs)	Frameshift in paired domain → premature stop	Heterozygous	Severe oligodontia affecting most molars and premolars	Autosomal dominant	[132,133,134]
c.1A>G (p.Met1Val)	Initiation codon loss; predicted translation failure	Heterozygous	Familial oligodontia and regional odontodysplasia; severe posterior tooth agenesis	Autosomal dominant	[135,136,137]
c.1A>C (p.Met1Leu)	Start-loss (initiation codon) variant at the 5′ end of PAX9; abolishes the canonical ATG start codon → predicted null allele / haploinsufficiency; upstream of the paired-domain–encoding region	Heterozygous	Familial isolated non-syndromic oligodontia, predominantly affecting posterior teeth (molars and premolars)	Autosomal dominant	[138,139]
c.2T>A (p.Met1Lys)	Start-loss (initiation codon) missense variant changing Met1 to Lys; prevents normal translation initiation → functional null allele; N-terminal region upstream of paired domain	Heterozygous	Non-syndromic familial oligodontia with marked posterior tooth agenesis	Autosomal dominant	[126,128]
c.59C>T (p.Pro20Leu)	Missense variant in the N-terminal portion of the paired domain (highly conserved region); functional studies show reduced DNA binding and transactivation (hypomorphic allele)	Heterozygous	Non-syndromic tooth agenesis, strongly associated with absence of multiple third molars (often ≥3 third molars missing); sometimes broader molar oligodontia	Likely autosomal dominant with variable expressivity	[123,140]
c.72_76dup (p.Arg26fs)	5 bp duplication in exon 2 causing a frameshift at Arg26 and premature stop codon in the paired domain → truncated protein, loss-of-function / haploinsufficiency	Heterozygous	Familial non-syndromic tooth agenesis with posterior-predominant oligodontia (molar agenesis)	Autosomal dominant	[133,141,142]
c.139C>T (p.Arg47Trp)	Missense substitution in the paired-box DNA-binding domain; disrupts conserved residue critical for transcriptional activation	Heterozygous	Non-syndromic oligodontia; severe tooth agenesis phenotype	Autosomal dominant	[143]
c.112C>T (p.Arg38Ter)	Nonsense in paired domain	Heterozygous	Non-syndromic oligodontia with molar and premolar agenesis	Autosomal dominant	[144]
c.792_793insC (p.Val265fs)	C-terminal frameshift → truncated transactivation region	Heterozygous	Non-syndromic oligodontia	Autosomal dominant	[145]

## Data Availability

No new data were created or analyzed in this study. Data sharing is not applicable to this article.
